# Associations among perceived health competence, effortful control, self-control, and personality traits in Japanese university students

**DOI:** 10.1038/s41598-023-29720-8

**Published:** 2023-02-13

**Authors:** Takuma Yoshioka, Kumiko Ohara, Katsumasa Momoi, Tomoki Mase, Harunobu Nakamura

**Affiliations:** 1 Graduate School of Human Development and Environment, Kobe University, 3-11 Tsurukabuto, Nada, Kobe, Hyogo 657-8501 Japan; 2 Department of Hygiene and Public Health, Kansai Medical University, 2-5-1 Shin-machi, Hirakata, Osaka 573-1010 Japan; 3 Faculty of Health and Welfare, Tokushima Bunri University, 180 Nishihama-hoji, Yamashiro-cho, Tokushima, Tokushima 770-8514 Japan; 4 Faculty of Human Development and Education, Kyoto Women’s University, 35 Kitahiyoshi-cho, Imakumano, Higashiyama-ku, Kyoto, Kyoto 605-8501 Japan

**Keywords:** Psychology, Human behaviour

## Abstract

Perceived health competence is thought to contribute to lifelong healthy behavior. However, the factors that affect perceived health competence have not been investigated. We investigated the associations among perceived health competence, effortful control, self-control, and personality traits in university students and proposed a model of how these factors affect perceived health competence. The participants were 320 Japanese university students who completed a questionnaire regarding their height, weight, perceived health competence, effortful control, self-control, and personality traits. The three-step multiple regression analysis showed that effortful control was positively associated with the perceived health competence, and self-control was positively with, and impulsivity was inversely associated with effortful control respectively, indicating that effortful control was an intermediate factor. Structural equation modeling showed a good fit for both genders, with a common path for both genders to perceived health competence via effortful control and a different involvement of personality traits for men and women. These results suggest that effortful control is directly associated with perceived health competence; in addition, both self-control and impulsiveness are indirectly associated with perceived health competence via effortful control.

## Introduction

The transition period from adolescence to young adulthood is considered to be an important period during which young people become independent in their decision-making and establish long-term health behavior patterns^1^. Additionally, the Healthy People 2030^2^ and Health Japan 21 (the Second Term)^3^ guidelines consider establishing healthy behaviors as an important goal of adolescence.

Practicing healthy behaviors relates to both perceived health competence and self-efficacy^4–7^. However, self-efficacy applies to for specific behaviors, whereas perceived health competence applies more generally to behaviors that individuals relate to their own health. Perceived health competence refers to an individual’s confidence in their ability to effectively manage their health outcomes, and is also related to self-efficacy^7^. Therefore, increasing perceived health competence is important for a lifetime of healthy living and requires an understanding of the factors that influence perceived health competence.

Perceived health competence, similar to self-efficacy, is related to perceived control beliefs in the area of health^7^, and refers to the degree to which an individual feels capable of effectively managing their health behaviors and health outcomes^7^. Therefore, it is possible that psychological factors regarding health control have a relationship with perceived health competence. Psychological factors related to health management include effortful control and self-control. Effortful control represents executive attention and is thought to be the basis of both inhibitory and initiatory control of behavior^8^. To the best of our knowledge, no previous research studies have examined direct associations between effortful control, self-control and perceived health competence. However, effortful control has been reported to be associated with actual health behaviors in previous studies^9–11^, suggesting that it is related to perceived health competence. In contrast, self-control is the pursuit of desirable and undesirable goals^12^. Self-control has been reported to be associated with various health behaviors^13–15^, suggesting that it may also be associated with perceived health competence. Personality is the dynamic organization of the psycho-physical systems within an individual that determine their characteristic behavior and thought patterns^16^. Thus, previous studies revealed that personality traits are related to health behaviors^17–19^, raising the possibility that personality traits have some influence on perceived health competence. The five-factor model of personality, referred to as the Big Five, is the predominant model for capturing and understanding individual differences in personality^20^. The five factor model consists of extraversion, agreeableness, conscientiousness, neuroticism, and openness to experience^20^. Recently, Gosling et al. developed the Ten-Item Personality Inventory (TIPI) as a brief and time-efficient measure of the five factor model personality domains^21^.

Taken together, these previous studies suggest that these psychological factors may be related to perceived health competence; however, their interrelationships have not yet been examined. Although effortful control and self-control are similar concepts, because effortful control includes behavioral aspects of self-control such as behavioral control and behavior initiation^22^, it is possible that effortful control is more directly related to perceived health competence. It has also been reported that self-control can be divided into the two dimensions of inhibition and impulsivity^23^. Previous research has demonstrated that self-control and impulsivity have a negative relationship, suggesting that impulsivity is more directly related to self-control. It is therefore possible that while effortful control may be directly related to perceived health competence, the other indicators may be indirectly related. The current study attempted to examine the direct and/or indirect relationships between perceived health competence and each indicator, and to create a model on the basis of these relationships.

## Methods

### Participants

Prior to the questionnaire survey conduction, inclusion/exclusion criteria were determined. Inclusion criteria for this study were undergraduate or graduate students, and the upper age limit was 29 years. No exclusion criteria were specified. In addition, prior-power analysis was conducted using G* Power software^24,25^ to calculate the required sample size with the following assumptions: a medium effect size of .15, an α of .05, and a power of .80. In addition, multiple linear regression, with eight independent variables, was used as a statistical test. On the basis of these assumptions, 109 participants were needed.

Participants were 352 undergraduate and graduate students who attended university classes in the Japanese cities of Kobe, Kyoto, and Tokushima. Survey data were collected in 2021, and participants did not receive remuneration. Anonymous, self-administered questionnaires were provided to 352 students. Of the 352 students, 320 returned valid questionnaire responses with all questions answered. Thus, the response rate, calculated by dividing the number of questionnaires with valid responses by the number of provided questionnaires, was 90.9% (n = 320, 105 men and 215 women, 19.5 ± 1.3 years old). All procedures performed in studies involving human participants followed the ethical standards of the institutional and national research committee and the 1964 Helsinki declaration and its later amendments. The study protocol was approved by the Human Ethics Committee of the Graduate School of Human Development and Environment, Kobe University (approval number, 504). All participants enrolled in this study provided informed consent.

### Questionnaires

Participants were asked about their height (cm), weight (kg), perceived health competence, impulsivity, personality traits, effortful control, and self-control. Body mass index (BMI) (kg/m^2^) was calculated by dividing body weight (kg) by the square of the height (m) for each participant.

Perceived health competence was measured using the Japanese version of the Perceived Health Competence Scale (PHCS)^26^, which was originally developed by Smith and colleagues^7^. The Japanese version of the PHCS consists of eight items, each of which is rated on a 5-point Likert scale, with response options ranging from 1 (“strongly disagree”) to 5 (“strongly agree”). The higher the score, the higher the individual’s awareness of perceived health competence. In our sample, the Cronbach’s α coefficient for the scale was .838.

Impulsivity was measured using the revised Japanese version of the ABbreviated Impulsiveness Scale (ABIS)^27^ which was originally developed by Coutlee and colleagues^28^. The original version of the ABIS was reconstructed from the 30-item Barratt Impulsiveness Scale (BIS-11)^29^ and developed as a shortened version of BIS-11 with a 13-item three factor structure. The Japanese version of the ABIS is a 13-item scale measuring attention impulsiveness (inability to concentrate) (5 items), non-planning impulsiveness or lack of future planning impulsiveness (lack of premeditation) (4 items), and motor impulsiveness (action without thought) (4 items), each of which is rated on a 6-point Likert scale, with response options ranging from 1 (“strongly disagree”) to 6 (“strongly agree”). Each subscale is scored by averaging responses on all the relevant subscale items, after accounting for the reverse-scored items. Higher scores indicate greater impulsivity. In this study, the Cronbach’s α for this scale was .796.

Personality traits were evaluated using the Japanese version of the Ten Item Personality Inventory (TIPI-J)^30^. TIPI was originally developed by Gosling et al.^21^ as an improved version of the five-factor model of personality^31^. The TIPI-J consists of 10 items, each of which is rated on a 7-point Likert scale, with response options ranging from 1 (“strongly disagree”) to 7 (“strongly agree”). The TIPI-J measures five important personality traits: extraversion, agreeableness, conscientiousness, neuroticism, and openness. The characteristics of five personality traits are as follows^32^. Extraversion is defined by positive emotions, such as gregariousness and the tendency to seek outstimulation. Agreeableness describes an individual’s level of cooperativeness and compassion. Conscientiousness refers to carefulness and organizational ability. Neuroticism includes negative emotions, such as anxiety and depression, and is commonly defined as emotional instability. Openness captures imagination and intellectual curiosity. It has adequate convergent and discriminant validity, test–retest reliability, and patterns of external correlates^30^. Cronbach’s α for extraversion, agreeableness, conscientiousness, neuroticism, and openness in this study were .716, .353, .510, .376, and .445, respectively.

To measure effortful control, we used the Japanese version of the Effortful Control Scale^33^, which was developed from the original Effortful Control Scale included in the Adult Temperament Questionnaire^8^. The Japanese version of the Effortful Control Scale consists of 35 items, each of which is rated on a 4-point Likert scale, with response options ranging from 1 (“Not applicable at all”) to 4 (“Very applicable”). It includes the following three subscales: attentional control which involves the ability to voluntarily focus or shift attention (12 items), inhibitory control which is the ability to effortfully inhibit behavior (11 items), and activation control which involves the ability to activate behavior even when one is not fully motivated (12 items)^34–36^. Cronbach’s α for this scale in this study was .873.

Self-control was measured using the Japanese version of the Brief Self Control Scale (BSCS-J)^37^ which was originally developed by Tangney et al.^38^. BSCS-J is a scale which measures an individual’s capacity to override or change their inner responses, as well as to interrupt undesired behavioral tendencies and to refrain from acting on them^37,38^. The BSCS-J consists of 13 items, each of which is rated on a 5-point Likert scale, with response options ranging from 1 (“Not applicable at all”) to 5 (“Very applicable”). Total scores range from 13 to 65 points: the higher the score, the more likely individuals are to control themselves. Cronbach’s α for this scale in this study was .834.

### Statistical analysis

Student’s t-tests were used for evaluation of the differences between men and women. Pearson’s correlation coefficients were calculated for the associations between BMI, perceived health competence, effortful control, self-control, impulsiveness, and personality traits.

We used the method described by Baron and Kenny^39^ and Ohara et al.^40^ for three-step multiple linear regression analysis to determine whether effortful control could be considered an intermediate variable. In the first step, perceived health competence was the dependent variable and the other indicators (except effortful control) were independent variables. In the second step, effortful control was the dependent variable, and the other indicators were independent variables. In the final step, perceived health competence was the dependent variable and the other indicators (including effortful control) were independent variables. In the results of the three-step analysis, effortful control was considered to be an intermediate factor if the following three conditions were satisfied; (1) self-control as an independent variable was significantly related to perceived health competence as a dependent variable in the first step when effortful control was not included in the model as an independent variable, (2) In the second step, self-control as an independent variable was significantly related to effortful control as a dependent variable, (3) In the final step, effortful control as an independent variable was significantly related to perceived health competence as a dependent variable when both self-control and effortful control were included in the model as independent variables.

The variance inflation factor (VIF) was used to detect the degree of multicollinearity among the variables. VIF > 10 was considered to be indicative of multicollinearity and should be excluded from the regression model. Effect sizes for the multiple regression analysis were reported as Cohen’s ƒ^2^ and interpreted according to Cohen’s recommendation of .02 for a small effect, .15 for a medium effect, and .35 for a large effect^41^. Structural equation modeling (SEM) was used to explore the association between perceived health competence and related factors. The fit indices used to evaluate the model were the ratio of the chi-square value to the degrees of freedom (χ^2^/df) < 2.0^42^; standardized root mean square residual (SRMR) < .08; root mean square error of approximation (RMSEA) including the 90% confidence interval < .05 (values between .05 and .08 were considered acceptable); and the comparative fit index (CFI) and Tucker-Lewis index (TLI) ≥ .90^43,44^. We also calculated Akaike’s information criterion (AIC) to estimate the relative quality of our model.

The level of statistical significance was set at .05. All statistical analyses were performed with SPSS^®^ 27.0 for Windows (International Business Machines Corp, Armonk, NY) and Amos 27.0 for Windows (International Business Machines Corp, Armonk, NY).

## Results

Height, weight, and BMI were significantly higher in men than in women. On the TIPI-J, extraversion was higher in women than in men. The other measures showed no significant gender differences (Table 1).

**Table 1:** Anthropometry, perceived health competence, effortful control, self-control, impulsiveness, and personality traits by gender.

	Men(n = 105)	Women(n = 215)	*p*-value^a^
Height (cm)	172.0 ± 5.3	159.0 ± 5.5	< .001
Body weight (kg)	62.6 ± 9.5	51.2 ± 6.1	< .001
BMI (kg/m^2^)	21.2 ± 3.0	20.2 ± 2.1	.005
Perceived health competence	3.1 ± 0.8	3.1 ± 0.7	.884
Effortful control			
Inhivitory control	2.7 ± 0.4	2.8 ± 0.5	.713
Activation control	2.6 ± 0.5	2.6 ± 0.5	.542
Attention control	2.3 ± 0.5	2.4 ± 0.5	.057
Total	2.5 ± 0.4	2.6 ± 0.4	.225
Self-control	36.2 ± 8.6	37.2 ± 8.4	.349
Impulsiveness			
Motor	2.9 ± 0.8	3.1 ± 0.9	.201
Non-planning	3.4 ± 0.9	3.3 ± 0.8	.396
Attentional	3.1 ± 0.7	3.1 ± 0.7	.525
Total	3.1 ± 0.6	3.1 ± 0.6	.935
Personality traits			
Extraversion	3.6 ± 1.5	4.0 ± 1.5	.016
Agreeableness	5.0 ± 1.1	5.1 ± 1.0	.263
Conscientiousness	3.2 ± 1.2	3.4 ± 1.2	.100
Neuroticism	4.4 ± 1.2	4.5 ± 1.1	.843
Openness	3.9 ± 1.3	3.8 ± 1.2	.262

Values are means ± standard deviations.

*BMI* Body mass index.

^a^*p*-value for Student’s t-test between men and women.

Pearson’s correlation coefficients for the associations between BMI, perceived health competence, effortful control, self-control, impulsiveness, and personality traits in men and women are shown in Tables 2 and 3, respectively. For both men and women, perceived health competence was significantly positively correlated with effortful control, self-control, extraversion, agreeableness, conscientiousness, and openness, and significantly inversely correlated with impulsiveness and neuroticism. Specifically, effortful control was significantly positively correlated with self-control, conscientiousness, and openness, and significantly inversely correlated with impulsiveness in men. In women, effortful control was significantly positively correlated with self-control, extraversion, agreeableness, conscientiousness, and openness, and significantly inversely correlated with BMI, impulsiveness, and neuroticism. Self-control was significantly positively correlated with extraversion, agreeableness, conscientiousness and openness, and significantly inversely correlated with impulsiveness and neuroticism in men. In women, self-control was significantly correlated with conscientiousness and openness, and significantly inversely correlated with impulsiveness.

**Table 2:** Pearson's correlation coefficients between perceived health competence, effortful control, self-control, BMI, impulsiveness, and personality traits in men.

	Perceived health competence	BMI	Effortful contorl	Self-control	Impulsiveness	Extraversion	Agreeableness	Conscientious- ness	Neuroticism	Openness
Perceived health competence	–									
BMI	.061	–								
Effortful control	.562*	.065	–							
Self-control	.549*	.016	.758*	–						
Impulsiveness	− .309*	− .101	− .596*	− .561*	–					
Extraversion	.211*	.111	.187	.257*	.027	–				
Agreeableness	.231*	.250*	.112	.215*	− .337*	.042	–			
Conscientiousness	.401*	.127	.499*	.684*	− .389*	.234*	.186	–		
Neuroticism	− .227*	.090	− .189	− .274*	.153	− .273*	− .195*	− .041	–	
Openness	.382*	− .031	.292*	.385*	− .185	.480*	.102	.288*	− .382*	–

n = 105.

*BMI* Body mass index.

*p < .05 (Pearson’s correlation coefficient).

**Table 3:** Pearson’s correlation coefficients between perceived health competence, effortful control, self-control, BMI, impulsiveness, and personality traits in women.

	Perceived health competence	BMI	Effortful contorl	Self-control	Impulsiveness	Extraversion	Agreeableness	Conscientious	Neuroticism-ness	Openness
Perceived health competence	–									
BMI	− .102	–								
Effortful control	.469*	− .173*	–							
Self-control	.411*	− .071	.793*	–						
Impulsiveness	− .337*	.061	− .641*	− .629*	–					
Extraversion	.321*	.021	.178*	.108	.037	–				
Agreeableness	.250*	− .036	.154*	.125	− .289*	− .127	–			
Conscientiousness	.312*	− .080	.517*	.629*	− .511*	.150*	.018	–		
Neuroticism	− .149*	.138*	− .190*	− .105	.046	− .057	− .073	− .054	–	
Openness	.214*	− .049	.291*	.282*	− .071	.316*	.030	.286*	− .098	–

n = 215.

*BMI* Body mass index.

*p < .05 (Pearson’s correlation coefficient).

The results of simple regression analysis are shown in Table 4, and those for multiple regression analyses in Table 5. We conducted multiple linear regression analysis in three steps as described by Baron and Kenny^39^ and Ohara et al.^40^ to verify if effortful control could be considered an intermediate variable (Table 5). The multiple linear regression results for Steps 1, 2, and 3 are shown in Table 5.

**Table 4:** Simple regression analysis with perceived health competence or effortful control as the dependent variable.

	Men (n = 105)		Women (n = 215)	
	β	*p*-value	β	*p*-value
Perceived health competence^a^				
Effortful control	.562	< .001	.469	< .001
Self-control	.549	< .001	.411	< .001
Impulsiveness	− .309	.001	− .337	< .001
Personality traits				
Extraversion	.211	.031	.321	< .001
Agreeableness	.231	.018	.250	< .001
Conscientiousness	.401	< .001	.312	< .001
Neuroticism	− .227	.020	− .149	.029
Openness	.382	< .001	.214	.002
Effortful control^a^				
Self-control	.758	< .001	.793	< .001
Impulsiveness	− .596	< .001	− .641	< .001
Personality traits				
Extraversion	.187	.056	.178	.009
Agreeableness	.112	.255	.154	.024
Conscientiousness	.499	< .001	.517	< .001
Neuroticism	− .189	.053	− .190	.005
Openness	.292	.002	.291	< .001

^a^Dependent variable.

**Table 5:** Multiple regression analysis with perceived health competence or effortful control as the dependent variable.

	Men (n = 105)			Women (n = 215)		
	β	*p*-value	VIF	β	*p*-value	VIF
[Step 1]^a^						
Self-control	.437	.002	2.664	.243	.005	2.207
Impulsiveness	.029	.782	1.653	− .101	.210	1.960
Personality traits						
Extraversion	− .015	.876	1.407	.311	< .001	1.148
Agreeableness	.117	.190	1.165	.223	< .001	1.143
Conscientiousness	.038	.745	2.029	.049	.526	1.823
Neuroticism	− .016	.861	1.321	− .081	.162	1.023
Openness	.197	.055	1.541	.011	.861	1.248
[Step 2]^b^						
Self-control	.635	< .001	2.664	.605	< .001	2.207
Impulsiveness	− .301	< .001	1.653	− .278	< .001	1.960
Personality traits						
Extraversion	.053	.469	1.407	.102	.014	1.148
Agreeableness	− .116	.083	1.165	.003	.948	1.143
Conscientiousness	− .042	.636	2.029	− .047	.363	1.823
Neuroticism	.020	.780	1.321	− .103	.009	1.023
Openness	− .002	.980	1.541	.071	.099	1.248
[Step 3]^c^						
Effortful control	.430	.001	2.719	.253	.014	3.251
Self-control	.164	.282	3.761	.091	.386	3.398
Impulsiveness	.159	.143	1.899	− .031	.715	2.211
Personality traits						
Extraversion	− .038	.683	1.414	.285	< .001	1.182
Agreeableness	.167	.054	1.202	.222	< .001	1.143
Conscientiousness	.056	.615	2.033	.061	.426	1.830
Neuroticism	− .025	.781	1.322	− .056	.341	1.057
Openness	.198	.043	1.541	− .007	.915	1.264

Step 1 : perceived health competence as the dependent variable.

Step 2 : effortful control as the dependent variable.

Step 3 : perceived health competence as the dependent variable and effortful control as the intermediate factor.

*VIF* Variance inflation factor.

^a^Adjusted R^2^ (Cohen's *f*^2^) of multiple regression: .302 (.433) for men, .297 (.422) for women.

^b^Adjusted R^2^ (Cohen's *f*^2^) of multiple regression: .606 (1.538) for men, .682 (2.145) for women.

^c^Adjusted R^2^ (Cohen's *f*^2^) of multiple regression: .369 (.585) for men, .314 (.458) for women.

In Step 1, where perceived health competence was entered into the model as the dependent variable and effortful control was not entered into the model as the independent variable, the multiple regression for men showed that self-control was significantly positively associated with perceived health competence. For women, self-control, extraversion, and agreeableness were significantly positively associated with perceived health competence.

In Step 2, where effortful control was entered into the model as the dependent variable and self-control was entered into the model as the independent variable, the multiple regression for men showed that self-control was significantly positively associated with effortful control, while impulsiveness was significantly inversely associated with effortful control. Among women, self-control and extraversion were significantly positively associated with effortful control, and impulsiveness and neuroticism were significantly inversely associated with effortful control.

In Step 3, in which perceived health competence was entered into the model as the dependent variable and both effortful control and self-control were entered into the model as the independent variables, effortful control and openness were significantly positively associated with perceived health competence, but self-control was not associated with perceived health competence in men. In women, effortful control, extraversion, and agreeableness were significantly positively associated with perceived health competence, but self-control was not associated with perceived health competence.

**Figure 1:**
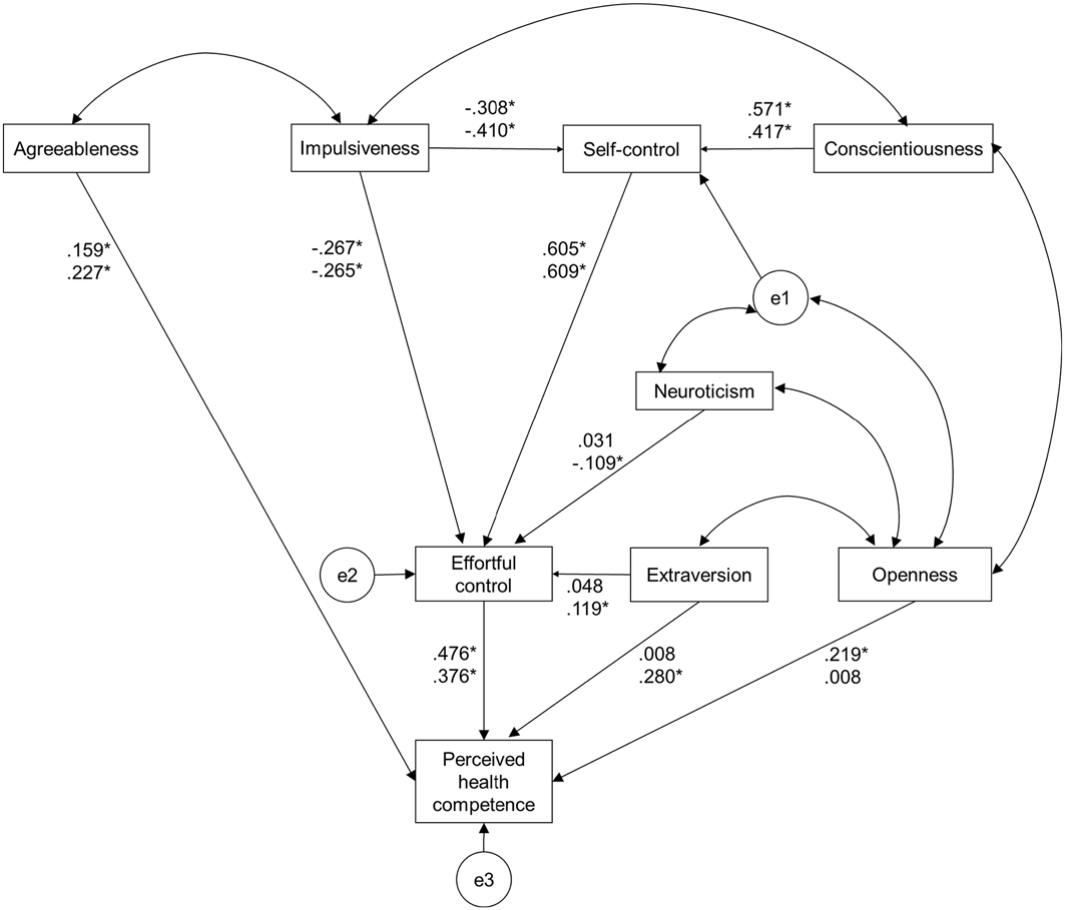
Associations between perceived health competence and effortful control, self-control, impulsiveness, and personality traits. Upper number is standardized coefficient for males and lower number is for females, and e1 through e3 stand for errors in each item. ^*^p < .05.

Figure 1 shows the results of the SEM and the relationships among perceived health competence, effortful control, self-control, impulsiveness, and personality traits. The model was finalized after inspection of the modification indices and allowing for the unique variances of five pairs to correlate. In men, self-control was significantly positively associated (*p* < .001), and impulsiveness was significantly inversely associated (*p* < .001), with effortful control. Effortful control, openness, and agreeableness were significantly positively associated with perceived health competence (effortful control, *p* < .001; openness, *p* = .010; agreeableness, *p* = .046). Conscientiousness was significantly positively associated with self-control (*p* < .001), while impulsiveness was significantly inversely associated with self-control (*p* < .001). The model showed a good fit: χ^2^ = 39.958, df = 19, χ^2^/df = 2.103; SRMR = .112; GFI = .927, AGFI = .826, TLI = .877, CFI = .935, and RMSEA = .103. The AIC was 91.958.

In women, self-control and extraversion were significantly positively associated (self-control, *p* < .001; extraversion, *p* = .002), and impulsiveness and neuroticism were significantly inversely associated (impulsiveness, *p* < .001; neuroticism, *p* = .005), with effortful control. Effortful control, agreeableness, and extraversion were significantly positively associated with perceived health competence (all *p* < .001). Conscientiousness was significantly positively associated with self-control (*p* < .001) and impulsiveness was significantly inversely associated with self-control (*p* < .001). The model showed a good fit: χ^2^ = 23.684, df = 19, χ^2^/df = 1.247; SRMR = .057; GFI = .976, AGFI = .944, TLI = .986, CFI = .992, and RMSEA = .034. The AIC was 75.684.

**Figure 2:**
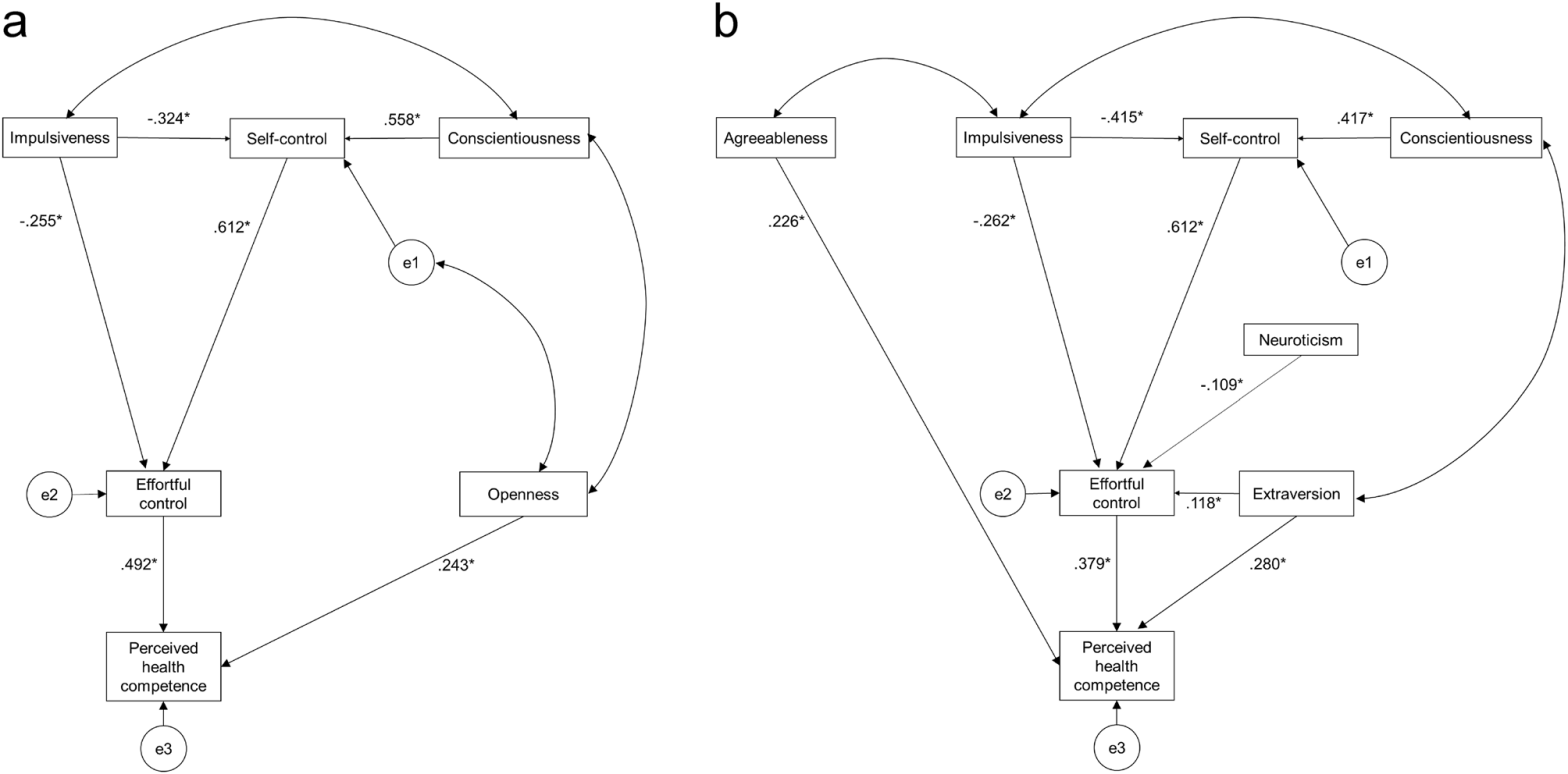
Associations between perceived health competence and effortful control, self-control, impulsiveness, and personality traits in men (**a**), and in women (**b**). The non-significant associations in structural equation modeling or multiple regression analysis for each gender were removed and the saturated models were determined. The number is standardized coefficient and e1 through e3 stand for errors in each item. ^*^p < .05.

Next, we removed the non-significant associations in SEM and multiple regression analysis for each gender and determined the saturated models. The model was finalized after inspection of the modification indices and allowing for the unique variances of the pairs to correlate. The model for men (Fig. 2a) showed a good fit: χ^2^ = 7.787, df = 6, χ^2^/df = 1.298; SRMR = .064; GFI = .976, AGFI = .916, TLI = .983, CFI = .993, and RMSEA = .054. The AIC was 37.787. The model for women (Fig. 2b) also showed a good fit: χ^2^ = 14.044, df = 16, χ^2^/df = .878; SRMR = .046; GFI = .984, AGFI = .964, TLI = 1.006, CFI = 1.000, and RMSEA = .000. The AIC was 54.044.

## Discussion

The aim of this study was to investigate the associations between perceived health competence and related psychological factors in university students. In addition, we proposed a model incorporating the various factors that affect perceived health competence.

The multiple linear regression analysis was then performed over three steps according to Baron and Kenny^39^ and Ohara et al.^40^. The three-step analysis indicated that effortful control was an intermediate variable. Furthermore, the effect sizes in the three-step multiple regression analysis were large, indicating that perceived health competence, effortful control, and self-control have firm associations.

Then, we developed a model of perceived health competence, effortful control, and related constructs, on the basis of the results of the three-step multiple regression analysis. The common model for men and women did not show a good fit, so we constructed models for men and women separately. The separate models for men and women showed a good fit, but some gender-related differences were shown as well as common paths.

The common path for men and women was that self-control was influenced by impulsivity and conscientiousness and was related to perceived health competence via effortful control. Prior research has reported a negative association between self-control and impulsivity^45^, as well as a positive association between self-control and conscientiousness^38^. In addition, a positive association between self-control and effortful control has also been reported, although the measures of self-control have differed from the present study^46^. Self-control is a broader personality trait that emerges over time, and is considered to be part of the foundation of effortful control^47,48^. Effortful control refers to the ability to self-regulate, or to actively control prevailing behaviors and emotional responses^49^. These might helpful for explaining our model, in which effortful control is more directly related to perceived health competence, while self-control, as the basis of effortful control, is related to perceived health competence via effortful control.

In the model, personality traits were also associated with a common path for men and women. In men, openness was directly related to perceived health competence, whereas in women, agreeableness and extraversion were related to perceived health competence. Marks et al.^6^ reported a positive correlation between perceived health competence and conscientiousness but a negative correlation between perceived health competence and neuroticism in the older people. Aside from Marks et al. study^6^, the relationship between perceived health competence and personality traits has rarely been investigated. Extraversion means actively working with others, having many friends, and having high social skills. Agreeableness represents cooperation in relationships; highly cooperative people are empathetic and supportive^32^, and show compassion and respect^50^. Openness refers to the ability to engage in intellectual thought; those with high openness are imaginative, interested in various things^32^, and also have intellectual curiosity and creativity. Conscientiousness shows productivity and a sense of responsibility^50^. In contrast, perceived health competence refers to the ability of individuals to be aware of and effectively manage their health^7^. In other words, perceived health competence requires individuals to understand their physical and mental condition, and then to select and execute the best health behaviors for themselves. Openness may help individuals understand health conditions. Extraversion and agreeableness are required to obtain a wider variety of information in selecting the best health behavior, and these personality traits are also important as behavior-enhancing factors, such as support for continuing health behaviors. These factors are in accord with the relationship between perceived health competence and personality traits observed in the current study. However, it is not clear why these relationships exhibit gender differences, because, to the best of our knowledge this is the first investigation of the relationships between perceived health competence and personality traits.

Regarding openness and agreeableness, the results of this study did not reveal gender differences. However, several previous studies, including large surveys and multinational collaborations, have demonstrated robust gender differences in agreeableness, with women generally scoring higher on these personality traits than men^51–55^. Agreeableness is a dimension associated with the maintenance of good interpersonal relationships and conflict avoidance^54^^,^^56^, and is a trait that may explain the tendency to engage in normative behavior, such as adherence to preventive actions. With regard to extraversion, previous studies have reported that women are more extraverted than men^30,40^. It has also been reported that higher extraversion is associated with desirable physical activity habits among women^57^. These previous findings support the association between perceived health competence and agreeableness among women revealed in the present study.

In addition, Oshio et al.^30^ conducted a survey of 902 university students in the process of creating TIPI-J, and found that women scored higher on extraversion than men, whereas men scored higher on openness than women. Conscientiousness, agreeableness, and neuroticism scores did not differ between men and women^30^. Ohara et al.^40^ also administered the TIPI to male and female college students and reported that extraversion and neuroticism were significantly higher in women and openness was significantly higher in men. In addition, according to Courtenay et al.^58^, men engage in riskier behaviors and hold riskier beliefs about their health than women. In addition, Evans et al.^59^ reported that the delayed onset of cancer symptoms and decreased self-examination among men may be caused by their low level of cancer awareness, poor knowledge of cancer warning signs, and relatively little contact with health professionals that may eliminate opportunities to promote cancer detection behaviors It has been suggested that this may be influenced by the following factors. Although openness was found to be directly related to perceived health competence among men in this study, it is precisely the level of health awareness and lack of knowledge among men that Evans et al.^59^ point to as being related to openness. Thus, we believe that differences in gender roles and responses to risk can be linked to gender differences in the relationship between personality traits and perceived health competence. These findings may relate to the gender differences in our models.

Some personality traits in TIPI-J in the current study did not have high Cronbach’s alpha values. This was also the case in Gosling’s study in which the TIPI was developed, where the Cronbach’ alpha values for the extraversion, agreeableness, conscientiousness, emotional stability, and openness to experience scales were .68, .40, .50, .73, and .45, respectively^21^. Gosling proposed the following reasons for this phenomenon^21^. Cronbach’s alpha is a function of mean inter-item correlation and the number of items comprising the scale. Therefore, in a multiple-item scale, a high Cronbach’s alpha value can be obtained by using multiple items with a high degree of content overlap. However, TIPI-J has only two items per scale. Thus, as a results of the emphasis on content validity, the inter-item correlations are lower than homogeneous scales. The relatively low inter-item correlations, combined with the fact that the TIPI has only two items per scale, results in unusually low internal consistency estimates. This discussion by Gosling^21^ is considered to be applicable to the present results. The low Cronbach’s alpha values for personality traits in the present study does not necessarily undermine the validity of this scale.

In recent years, various reports of mental health deterioration among college students were made during lockdowns and other behavioral restrictions that were implemented as a result of the coronavirus disease 2019 (COVID-19) pandemic^60–63^. It was also reported that having a higher perception of health risk and being a woman are associated with a higher number and greater severity of negative feelings^60–63^. Lockdown and behavioral restriction require effortful restraint of behavior. Because this is precisely the behavior that corresponds to inhibitory control in effortful control^34–36^, it can be addressed by inhibitory control in effortful control. Therefore, even in situations where behavior is restricted because of the COVID-19 pandemic, it may be possible to reduce the impact on mental health by making efforts to increase effortful control. However, in the results of the current study, personality traits such as extraversion, agreeableness, and conscientiousness were related to paths to perceived health competence for women. Because the path to perceived health competence may not function in women who do not have high levels of these personality traits, it may be necessary to enhance social support.

Several limitations were involved in the present study. First, all of the participants were Japanese university students, recruited from a restricted area in Japan. Although it is possible that the present results can be adapted to subjects other than those in the current study if they have similar characteristics, further research will be required to verify whether the present results can be generalized. Second, because of the cross-sectional design used in this study, we do not have information regarding the temporal relationships among factors and cannot directly prove causality. In contrast, intermediate factors are assumed to have a causal relationship. Therefore, in the case of a cross-sectional study such as the present study, it is necessary to estimate whether a factor is an intermediate factor or not. Thus, we used methods described by Ohara et al.^40^ and Baron and Kenny^39^ to identify intermediate factors. Third, height and body weight were self-reported which could potentially introduce information bias and lead to misclassification of BMI. However, the BMI in the present study (men: 21.2 kg/m^2^; women: 20.2 kg/m^2^) were similar to those of the National Health and Nutrition Survey (15–19-year-old and 20–29 year-ole men: 21.1 and 22.9 kg/m^2^; 15–19-year-old and 20–29-year-old women: 20.2 and 21.0 kg/m^2^)^64^.

Finally, the results of this study indicate that perceived health competence among university students is related to individual psychological factors such as impulsivity, personality traits, effortful control, and self-control, and that the relationship between perceived health competence and psychological factors differs depending on gender. The findings obtained from the current study are thought to contribute to the provision of guidance suitable for individuals in health education for university students, taking gender, individual characteristics, and self-management ability into consideration.

## Conclusions

In the present study, we attempted to elucidate the associations among factors related to perceived health competence and psychometric factors in Japanese university students. The results indicated that effortful control is directly related to perceived health competence, and plays a role as an intermediate factor in the association between perceived health competence and self-control. In addition, conscientiousness is associated with self-control, and impulsiveness is associated with both self-control and effortful control. In women, agreeableness and extraversion are associated with perceived health competence, while in men, openness is associated with perceived health competence. These findings indicate that there is a common path for men and women to perceived health competence via effortful control, but that personality traits may impact perceived health competence differently for men and women.

## Data Availability

The data that support the findings of this study are available from the corresponding author upon reasonable request.
